# Catastrophic health expenditure on acute coronary events in Asia: a prospective study

**DOI:** 10.2471/BLT.15.158303

**Published:** 2016-01-28

**Authors:** Stephen Jan, Stephen W-L Lee, Jitendra PS Sawhney, Tiong K Ong, Chee Tang Chin, Hyo-Soo Kim, Rungroj Krittayaphong, Vo T Nhan, Yohji Itoh, Yong Huo

**Affiliations:** a George Institute for Global Health, King George V Building, 83–117 Missenden Road, Camperdown, NSW 2050, Australia.; b Department of Medicine, Queen Mary Hospital, Hong Kong Special Administrative Region, China.; c Department of Cardiology, Sir Ganga Ram Hospital, New Delhi, India.; d Department of Cardiology, Sarawak General Hospital, Kuching, Malaysia.; e National Heart Centre Singapore, Singapore.; f Clinical Research Institute, Seoul National University Hospital, Seoul, Republic of Korea.; g Department of Medicine, Siriraj Hospital, Bangkok, Thailand.; h Department of Medicine, Cho Ray Hospital, Ho Chi Minh City, Viet Nam.; i Clinical Science Division, AstraZeneca, Osaka, Japan.; j Department of Cardiology, Peking University First Hospital, Beijing, China.

## Abstract

**Objective:**

To estimate out-of-pocket costs and the incidence of catastrophic health expenditure in people admitted to hospital with acute coronary syndromes in Asia.

**Methods:**

Participants were enrolled between June 2011 and May 2012 into this observational study in China, India, Malaysia, Republic of Korea, Singapore, Thailand and Viet Nam. Sites were required to enrol a minimum of 10 consecutive participants who had been hospitalized for an acute coronary syndrome. Catastrophic health expenditure was defined as out-of-pocket costs of initial hospitalization > 30% of annual baseline household income, and it was assessed six weeks after discharge. We assessed associations between health expenditure and age, sex, diagnosis of the index coronary event and health insurance status of the participant, using logistic regression models.

**Findings:**

Of 12 922 participants, 9370 (73%) had complete data on expenditure. The mean out-of-pocket cost was 3237 United States dollars. Catastrophic health expenditure was reported by 66% (1984/3007) of those without insurance versus 52% (3296/6366) of those with health insurance (*P* < 0.05). The occurrence of catastrophic expenditure ranged from 80% (1055/1327) in uninsured and 56% (3212/5692) of insured participants in China, to 0% (0/41) in Malaysia.

**Conclusion:**

Large variation exists across Asia in catastrophic health expenditure resulting from hospitalization for acute coronary syndromes. While insurance offers some protection, substantial numbers of people with health insurance still incur financial catastrophe.

## Résumé

**Objectif:**

Estimer les coûts directs ainsi que l'incidence des dépenses de santé catastrophiques pour les personnes admises à l'hôpital avec un syndrome coronarien aigu en Asie.

Les participants ont été inscrits à cette étude par observation entre juin 2011 et mai 2012 en Chine, en Inde, en Malaisie, en République de Corée, à Singapour, en Thaïlande et au Viet Nam. Les sites devaient recruter au minimum 10 participants consécutifs ayant été hospitalisés pour un syndrome coronarien aigu. Les dépenses de santé catastrophiques ont été définies comme les coûts directs d'hospitalisation initiale > 30% du revenu annuel de référence des ménages, et ont été estimées six semaines après la sortie de l'hôpital. Nous avons évalué les associations entre les dépenses de santé et l'âge, le sexe, le diagnostic de l'affection coronarienne en question et la couverture d'assurance maladie des participants, à l'aide de modèles de régression logistique.

Sur les 12 922 participants, 9370 (73%) disposaient de données complètes sur les dépenses. Les coûts directs moyens s'élevaient à 3237 dollars des États-Unis. Des dépenses de santé catastrophiques ont été rapportées par 66% (1984/3007) des personnes sans assurance contre 52% (3296/6366) des personnes ayant une assurance maladie (P < 0,05). L'occurrence de dépenses de santé catastrophiques allait de 80% (1055/1327) des participants non assurés et 56% (3212/5692) des participants assurés en Chine, à 0% (0/41) en Malaisie.

**Conclusion:**

Les pays d'Asie présentent de gros écarts en matière de dépenses de santé catastrophiques suite à une hospitalisation pour des syndromes coronariens aigus. Si le fait d'être assuré offre une certaine protection, un grand nombre de personnes ayant une assurance maladie font encore face à des catastrophes financières.

## Resumen

**Objetivo:**

Estimar los costes directos y la incidencia del gasto sanitario catastrófico en las personas admitidas en hospitales que sufren síndromes coronarios agudos en Asia.

**Métodos:**

Los participantes se inscribieron entre junio de 2011 y mayo de 2012 en este estudio de observación en China, India, Malasia, la República de Corea, Singapur, Tailandia y Vietnam. Se solicitó a cada país que inscribiera un mínimo de 10 participantes consecutivos que hubieran sido hospitalizados por un síndrome coronario agudo. El gasto sanitario catastrófico se definió como costes directos de hospitalización inicial > 30% de los ingresos familiares anuales de referencia, y se evaluó seis semanas después de recibir el alta hospitalaria. Se evaluó la relación entre el gasto sanitario y la edad, el sexo, el diagnóstico del caso coronario y la situación del seguro sanitario del participante, mediante modelos de regresión logística.

**Resultados:**

De 12 922 participantes, 9 370 (73%) tenían datos completos sobre el gasto. El coste directo medio fue de 3 237 dólares estadounidenses. El gasto sanitario catastrófico se registró en un 66% (1 984/3 007) de aquellos pacientes que no contaban con seguro, frente a un 52% (3 296/6 366) de los que contaban con seguro (P < 0,05). La aparición de gastos catastróficos variaba de un 80% (1 055/1 327) de participantes sin seguro y un 56% (3 212/5 692) de participantes asegurados en China a un 0% (0/41) en Malasia.

**Conclusión:**

Existe una gran variación en Asia en lo referente al gasto sanitario catastrófico derivado de la hospitalización por síndromes coronarios agudos. Aunque los seguros ofrecen cierta protección, existe un gran número de personas con seguro sanitario que aún incurren en catástrofe financiera.

## ملخص

**الغرض:**

تقدير التكاليف المدفوعة من جانب المريض وأسرته ووقوع حالات من الإنفاق الصحي الباهظ للأشخاص الذين يدخلون المستشفى لإصابتهم بمتلازمات الشريان التاجي الحادة في آسيا.

**الطريقة:**

تم تسجيل المشاركين في الفترة من يونيو/حزيران عام 2011 ومايو/أيار عام 2012 في هذه الدراسة الرصدية في الصين والهند وماليزيا وجمهورية كوريا وسنغافورة وتايلند وفييت نام. وكانت المواقع ملزمة بتسجيل ما لا يقل عن 10 مشاركين عولجوا على التوالي بالمستشفى لإصابتهم بمتلازمة الشريان التاجي الحادة. وقد تم تعريف النفقات الصحية الباهظة بأنها التكاليف المدفوعة من جانب المريض وأسرته عند الدخول إلى المستشفى لتلقي العلاج أول مرة > 30% من الدخل السنوي للأسرة عند خط الأساس، وتم تقييم هذه النفقات بمرور ستة أسابيع بعد الخروج من المستشفى. قمنا بتقييم علاقات الاقتران بين النفقات الصحية والعمر والجنس وتشخيص مؤشر حالة الشريان التاجي ووضع التأمين الصحي للمشارك، وذلك باستخدام نماذج التحوف اللوجيستي.

**النتائج:**

من بين 12,922 مشاركًا، كان لدى 9370 مشاركًا (بنسبة 73%) البيانات الكاملة عن النفقات. بلغ متوسط النفقات المدفوعة من جانب المريض أو أسرته 3237 دولارًا أمريكيًا. وتم الإبلاغ عن النفقات الصحية الباهظة من جانب الأشخاص الذين لا يغطيهم التأمين الصحي بنسبة 66% (1984/3007) في مقابل أولئك الأشخاص الذين يغطيهم التأمين الصحي بنسبة 52% (3296/6366) (الاحتمال < 0.05). تراوح معدل حدوث النفقات الباهظة بنسبة تبلغ 80% (1055/1327) عند الأشخاص غير المؤمن عليهم وبنسبة 56% (3212/5692) عند المشاركين المؤمن عليهم في الصين، إلى 0% (0/41) في ماليزيا.

## 摘要

**目的:**

旨在评估亚洲急性冠心病住院病人灾难性卫生支出的自费费用和发生率。

**方法:**

2011 年 6 月至 2012 年 5 月间，受访者报名参加本次在韩国、马来西亚、泰国、新加坡、越南、印度和中国开展的观察性调查。每个调查地至少需 10 名可连续参与的受访者，且其曾因急性冠心病入院治疗。灾难性卫生支出指初次住院治疗的自费部分超出家庭年收入基准的 30%，且其在费用报销六个星期后予以评估。我们通过逻辑回归模型，评估了卫生支出与年龄、性别、冠心病诊断指标和受访者医疗保险状态之间的联系。

**结果:**

12 922 名受访者中，9370 (73%) 具备完整的支出数据。自费平均费用为 3237 美元。其中，未参保的受访者中，66% (1984/3007) 报告称发生灾难性卫生支出，而参加医疗保险的受访者中，52% (3296/6366) 报告称发生灾难性卫生支出 (P < 0.05)。灾难性支出发生率从中国未参保的 80% (1055/1327) 和参保的 56% (3212/5692) 到马来西亚的 0% (0/41)。

**结论:**

急性冠心病治疗引起的灾难性卫生支出在亚洲地区存在很大差异。尽管保险提供某种保障，但大批参加医疗保险的人群仍旧会出现重大财务问题。

## Резюме

**Цель:**

Подсчитать выплаты из собственных средств пациентов и долю катастрофических расходов на здравоохранение среди людей, госпитализированных с острым коронарным синдромом, в Азии.

**Методы:**

Участники были включены в обсервационное исследование в период с июня 2011 г. по май 2012 г. во Вьетнаме, Индии, Китае, Малайзии, Республике Корея, Сингапуре и Таиланде. Для исследования как минимум 10 участников подряд, госпитализированных по причине острого коронарного синдрома, понадобились специальные центры. Катастрофические расходы на здравоохранение составили выплаты из собственных средств пациентов при первоначальной госпитализации, превышающие 30% базового уровня годового дохода семьи. При этом оценка проводилась через 6 недель после выписки из стационара. Была проведена оценка взаимосвязи между расходами на здравоохранение и возрастом, полом, показателями диагностирования индексного коронарного синдрома и наличием медицинской страховки пациента с помощью моделей логистической регрессии.

**Результаты:**

Из 12 922 участников 9 370 (73%) предоставили полные данные о расходах. В среднем выплаты из собственных средств пациентов составили 3 237 долларов США. О катастрофических расходах на здравоохранение сообщили 66% (1 984 из 3 007) пациентов из тех, у кого медицинская страховка отсутствовала, против 52% (3 296 из 6 366) пациентов из тех, кто обладал медицинской страховкой (P<0,05). Частотность катастрофических расходов варьировалась от 80% (1 055 из 1 327) среди незастрахованных и 56% (3 212 из 5 692) застрахованных участников в Китае до 0% (0 из 41) в Малайзии.

**Вывод:**

Существует большая разница между странами Азии в отношении катастрофических расходов на здравоохранение, вызванных госпитализацией при остром коронарном синдроме. Несмотря на то что страхование предоставляет определенную защиту, значительное количество людей, имеющих медицинскую страховку, терпят финансовый крах в вопросах здравоохранения.

## Introduction

Acute coronary syndromes are caused by sudden, reduced blood flow to the heart muscle. These conditions are a major cause of mortality and morbidity in the Asia-Pacific region and account for around half of the global burden from these conditions, i.e. around seven million deaths and 129 million disability-adjusted life years (DALYs) annually from 1990 to 2010.^1^^–^^5^ Significantly, during this period associated mortality and morbidity accounted for nearly two-thirds of all DALYs and over half of deaths from acute coronary syndromes occurring in low- and middle-income countries.^5^ The management of acute coronary syndromes varies widely between countries in Asia. In this area, hospital admission can create significant financial hardships for participants as treatment costs in many settings are borne largely out-of-pocket.^1^^,^^2^^,^^6^^–^^8^ In India, for example, survey data indicate that household expenditure on health care is 16.5% higher in households where one or more adults has cardiovascular disease.^9^ In China, out-of-pocket costs for medications treating high blood pressure – a risk factor for acute coronary syndrome – limit the adherence to the treatment.^10^ In addition, out-of-pocket costs for health care act as a barrier to improvements in the treatment of acute coronary syndromes in hospitals.^11^

This economic burden on households due to out-of-pocket costs is commonly defined as catastrophic health expenditure when out-of-pocket expenditure over a year exceeds a certain threshold. This threshold has been defined in many ways: one of the most common definitions is out-of-pocket costs exceeding 30% of annual household income.^12^^–^^15^

There is a lack of evidence on the household economic burden associated with acute coronary syndromes in Asia. Studies that have considered the issue have been small-scale and localized^1^ or have studied cardiovascular disease in general^14^ rather than specific diseases.^16^ In China, India and the United Republic of Tanzania, more than 50% of people admitted with cardiovascular disease experienced catastrophic health expenditure.^14^ Among 210 people with acute coronary syndromes in Kerala, India, 84% (176/210) experienced catastrophic health expenditure based on a slightly different threshold of > 40% of disposable income (income minus expenditure on food).^2^

While health insurance potentially provides protection from the burden of out-of-pocket costs, the extent of such protection will vary across different health-care systems. Catastrophic health expenditure was reported to be more frequent in uninsured than insured participants with: stroke in China;^13^ cardiovascular disease in India;^14^ and injury in Viet Nam.^17^ There is some contrary evidence that health insurance coverage is associated with catastrophic health expenditure in China and Viet Nam.^7^^,^^18^ This may be attributed to insurance-based funding making treatment available to groups who may otherwise have not sought care but who, because of limited reimbursement, also incur high levels of out-of-pocket costs.^19^

Here we examine the out-of-pocket costs of hospitalization for acute coronary syndromes in China, Hong Kong Special Administrative Region (SAR) of China, India, Malaysia, the Republic of Korea, Singapore, Thailand and Viet Nam. We also assess the incidence of catastrophic health expenditure associated with such hospitalization and the influence of health insurance and other background characteristics on such outcomes.

## Methods

### Setting

The seven countries included in the study have a combined population of around 2.8 billion people (64% of the overall Asian population and 40% of the global population) and represent a mix of income categories and health-care systems. China, Hong Kong SAR, Republic of Korea and Singapore have high incomes; China, Malaysia and Thailand are upper-middle income countries; while India and Viet Nam are lower-middle income countries. Table 1 provides basic demographic, economic and disease indicators for each of the seven countries included in this study.

**Table 1:** Health system indicators for seven countries in Asia

Variable	China	China, Hong Kong SAR	India	Malaysia	Republic of Korea	Singapore	Thailand	Viet Nam
Population in 2013 (millions)^a^	1357	NA	1252	29.7	50.2	5.4	67.0	89.7
GNI/capita in 2013 (US$)^a^	6560 (UMI)	NA (HI)	1570 (LMI)	10 430 (UMI)	25 920 (HI)	54 040 (HI)	5340 (UMI)	1740 (LMI)
Health expenditure per capita in 2012 (US$)^a^	322	NA	61	410	1703	2426	215	102
Out-of-pocket expenditure as % of private health expenditure in 2012^a^	78.0	NA	86.0	79.0	79.1	93.9	55.8	85.0
Life expectancy from birth in 2012, years^a^	75	NA	66	75	81	82	74	76
Age-standardized CVD mortality in 2000–2012 (per 100 000 population)^21^	300	NA	306	296	92	108	184	193

CVD: cardiovascular disease; GNI: gross national income; HI: high income; LMI: lower-middle income; NA: not applicable; UMI: upper-middle income; US$: United States dollars.

^a^ World Bank income classification.^20^

Hong Kong SAR of China, Malaysia, the Republic of Korea, Singapore and Thailand have achieved universal health coverage, albeit through a varied combination of financing mechanisms.^22^^,^^23^ The health services are mainly provided by the public sector and health insurance generally plays a supplementary role in which participants access coverage mainly for private sector services or elective treatments. In China,^19^ India^24^ and Viet Nam^25^ there are known to be gaps in financial protection and heavy reliance on out-of-pocket payments in access to health care.

### EPICOR Asia study

The EPICOR Asia study^26^ is a prospective observational study of consecutively recruited participants surviving hospitalization for acute coronary syndromes, enrolled in 218 hospitals in seven countries in Asia between June 2011 and May 2012.

Participants were eligible for inclusion in the study if they were 18 years or older; hospitalized within 48 hours of symptom onset of the index event; with a discharge diagnosis of an acute coronary syndrome; provided written informed consent at discharge; and completed a contact order form agreeing to be contacted for regular follow-up interviews after discharge.

Participants were excluded if their acute coronary event was caused by, or was a complication of, surgery, trauma, gastrointestinal bleeding or post-percutaneous coronary intervention; hospitalization for other reasons; a condition or circumstance arose that in the opinion of the investigator could significantly limit follow-up; they were participating in a randomized interventional clinical trial; or they had concomitant serious/severe comorbidities, which at the discretion of the investigator might have limited short-term life expectancy.^26^

Participants were followed-up via telephone interviews at six weeks and three months after the index event, and subsequently every three months until 24 months following hospital discharge. Only baseline and six-week data are reported here as the focus of the study is on the economic burden associated with hospitalization for a relevant acute episode of acute coronary syndromes. Baseline data were collected through interviews with participants on: (i) demography; (ii) index event type (ST elevation myocardial infarction, non-ST elevation myocardial infarction, or unstable angina); and (iii) health insurance status (government, private, employer-provided, other or none). Further details of the data collection have been published previously.^26^

The study was conducted in compliance with the principles of the Declaration of Helsinki, International Conference on Harmonisation Good Clinical Practice guidelines and applicable legislation on non-interventional studies in participating countries. The protocol, including the informed consent form, was approved in writing by the applicable ethics committee of the participating centres according to local regulations in each country. The ethics committee also approved any other non-interventional study documents, according to local regulations. A list of participating centres is available from the corresponding author.

Health insurance status was defined as a binary variable based on whether individuals nominated any one of the listed forms of health insurance or none. Treatment costs associated with hospitalization, amount reimbursed and out-of-pocket costs were assessed at the follow-up interviews at six weeks after discharge and converted into United States dollars (US$) based on exchange rates in March 2013. The primary outcome, catastrophic health expenditure, was assessed on the basis of whether a participant had incurred out-of-pocket treatment costs greater than 30% of annual baseline household income.^12^^–^^15^

A multivariable logistic regression model was used to assess associations between catastrophic health expenditure and age, sex, the type of index event and health insurance status. The results are presented as odds ratios (OR) and corresponding 95% confidence intervals (CI).

Analyses were undertaken using SAS version 8.2 or later (SAS Institute, Cary, United States of America).

## Results

**Fig. 1:**
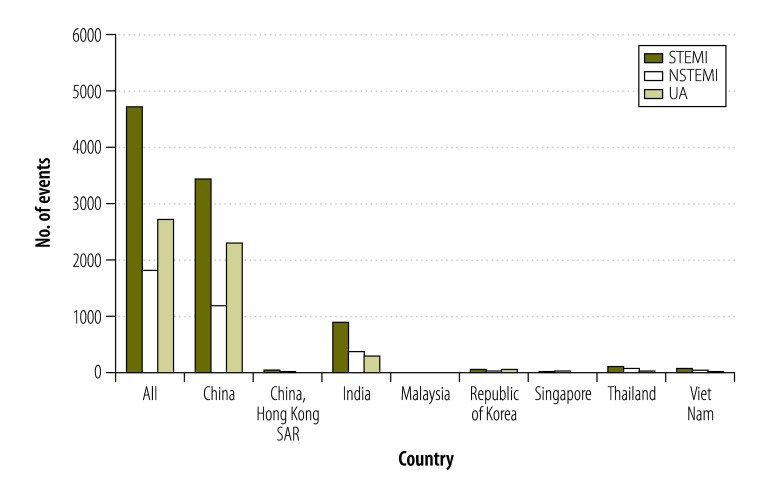
Number of events of acute coronary syndrome in participants enrolled between June 2011 and May 2012, in seven countries in Asia

NSTEMI: non-ST segment elevation myocardial infarction; STEMI: ST segment elevation myocardial infarction; UA: unstable angina.

Overall, 9370 out of 12 922 participants (73%) had complete economic data and were included in the analysis. Background characteristics of participants by health insurance status are shown in Table 2. The mean age of participants was 60 years and 77% (7209) were male. The majority of participants (7016) were from China (100 centres) where 61% (4266) had health insurance. There were 74 participants from Hong Kong SAR of China (three centres, 30% [22] insured), 1635 participants from India (41 centres, 56% [913] insured), 41 participants from Malaysia (two centres, 0% [0] insured), 169 from the Republic of Korea (11 centres, 12% [21] insured), 57 from Singapore (one centre, 18% [10] insured), 234 from Thailand (10 centres, 6% [15] insured), and 144 from Viet Nam (seven centres, 22% [32] insured). In terms of final diagnosis of index event, 51% (4762) had ST elevation myocardial infarction, 20% (2776) had non-ST elevation myocardial infarction and 29% (1832) had unstable angina. Fig. 1 indicates the number of events in each category across all seven countries. Of the 4762 participants who suffered a myocardial infarction with ST elevation, the majority (2854) were insured, while for participants who suffered from myocardial infarction without an ST elevation or unstable angina, around half of participants were uninsured; 48% (873/1832) and 47% (1310/2776), respectively.

**Table 2:** Baseline characteristics of participants, enrolled between June 2011 and May 2012, by health insurance status, in seven countries in Asia

Characteristic	Health insurance		
	Yes (*n* = 5279)	No (*n* = 4091)	Total (*n* = 9370)
**Age, average years (SD)**	60 (11)	61 (12)	60 (12)
**Age group, no. (%)**			
< 55 years	1660 (31)	1251 (31)	2911 (31)
55–64 years	1789 (34)	1328 (33)	3117 (33)
65–74 years	1311 (25)	933 (23)	2244 (24)
>75 years	519 (10)	579 (14)	1098 (12)
**Male, no. (%)**	4094 (77.6)	3115 (76.1)	7209 (76.9)
**Country, no. (%)**			
China	4266 (60.8)	2750 (39.2)	7016 (100.0)
China, Hong Kong SAR	22 (29.7)	52 (70.3)	74 (100.0)
India	913 (55.8)	722 (44.2)	1635 (100.0)
Malaysia^a^	0 (0.0)	41(100.0)	41 (100.0)
Republic of Korea	21 (12.4)	148 (87.6)	169 (100.0)
Singapore	10 (17.5)	47 (82.5)	57 (100.0)
Thailand	15 (6.4)	219 (93.6)	234 (100.0)
Viet Nam	32 (22.2)	112 (77.8)	144 (100.0)
**Place of residence, no. (%)**			
Rural	2008 (38.0)	1086 (26.5)	3094 (33.0)
Urban	3271 (62.0)	3005 (73.5)	6276 (67.0)
**Final diagnosis of index admission, no. (%)**			
STEMI	2854 (54.1)	1908 (46.6)	4762 (50.8)
NSTEMI	959 (18.2)	873 (21.3)	1832 (19.6)
UA	1466 (27.8)	1310 (32.0)	2776 (29.6)

NSTEMI: non-ST segment elevation myocardial infarction; STEMI: ST segment elevation myocardial infarction; SD: standard deviation; UA: unstable angina.

^a^ In Malaysia, services provided through the public sector are heavily subsidized -– responses regarding insurance status pertain to supplementary private cover.

**Fig. 2:**
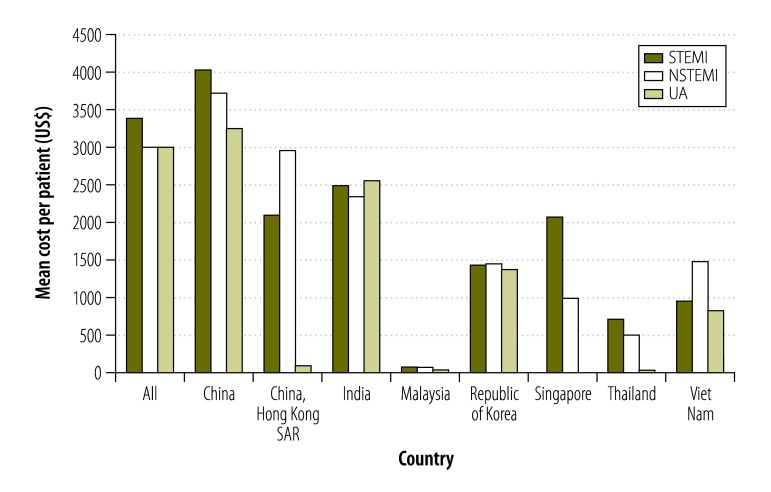
Out-of-pocket costs for participants, enrolled between June 2011 and May 2012, who suffered from acute coronary syndrome, in seven countries in Asia

NSTEMI: non-ST segment elevation myocardial infarction; STEMI: ST segment elevation myocardial infarction; UA: unstable angina; US$: United States dollars.

Note: National currencies were converted into US$ using the exchange rates of March 2013.

**Fig. 3:**
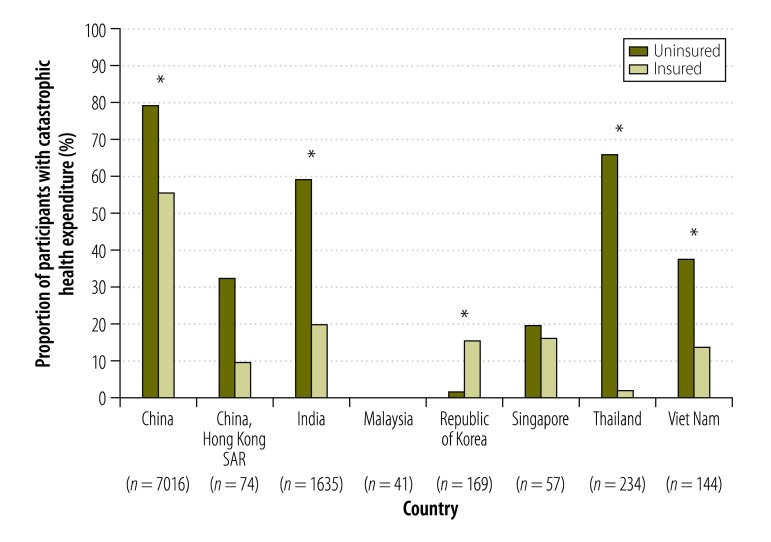
Catastrophic health expenditure for participants, enrolled between June 2011 and May 2012, who suffered from acute coronary syndrome, in seven countries in Asia

The total mean cost of hospitalization per participant was US$ 6478 for ST elevation myocardial infarction, US$ 5904 for non-ST elevation myocardial infarction and US$ 6026 for unstable angina. Average out-of-pocket costs of hospitalization were US$ 3421 for ST elevation myocardial infarction, US$ 3050 for non-ST elevation myocardial infarction and US$ 3052 for unstable angina. There was a broad range of out-of-pocket costs both by country and by index event type: for example, in Malaysia, mean out-of-pocket costs were US$ 69 for ST elevation myocardial infarction and US$ 72 for non-ST elevation myocardial infarction, while in China, mean out-of-pocket costs were US$ 4047 for ST elevation myocardial infarction, US$ 3743 for non-ST elevation myocardial infarction and US$ 3273 for unstable angina (Fig. 2). Catastrophic health expenditure was reported by 56% (5280/9373) of participants; of these, there was a significantly greater proportion in occurrence of this outcome amongst the uninsured (66%; 1984/3007) compared with insured (52%; 3296/6366; *P* < 0.05). The occurrence of catastrophic expenditure ranged from 80% (1055/1327) in uninsured and 56% (3212/5692) of insured participants in China, to 0% (0/41) in Malaysia (Fig. 3). In short, there was a broad range in the level of out-of-pocket costs and occurrence of catastrophic health expenditures experienced across the seven countries.

* *P* < 0.05.

Note: Catastrophic health expenditure was defined as out-of-pocket treatment costs greater than 30% of annual baseline household income. In Malaysia, services provided through the public sector are heavily subsidized and responses regarding insurance status pertain to supplementary private cover.

In the adjusted analysis, catastrophic health expenditure was significantly less likely for those with health insurance compared to those without insurance; OR: 0.563 (95% CI: 0.51–0.62). Catastrophic health expenditure was significantly more likely among older participants (Table 3).

**Table 3:** Factors associated with catastrophic health-care expenditure after an acute coronary syndrome event, for participants enrolled between June 2011 and May 2012, in seven countries in Asia

Factor	OR (95% CI)
**Age group, years**	
55–64 vs < 55	1.07 (0.97–1.19)
65–74 vs < 55	1.15 (1.02–1.28)
≥ 75 vs < 55	0.76 (0.66–0.88)
**Final diagnosis of index admission**	
NSTEMI vs STEMI	0.74 (0.66–0.82)
UA vs STEMI	0.78 (0.71–0.86)
**Gender**	
Female vs male	1.01 (0.91–1.12)
**Health insurance**	
Yes vs no	0.56 (0.51–0.62)

CI: confidence interval; NSTEMI: non-ST segment elevation myocardial infarction; OR: odds ratio; STEMI: ST segment elevation myocardial infarction; UA: unstable angina.

## Discussion

This study shows that the burden of out-of-pocket costs associated with treatment for acute coronary syndromes in Asia can be substantial, reflecting the limited financial protection available for hospitalization for these conditions. It further highlights large variation across countries in rates of catastrophic health expenditure resulting from hospitalization for acute coronary syndromes, and high rates of financial catastrophe incurred, particularly in China and India. With data from over 9000 respondents, we have conducted the largest ever prospective observational study of household economic burden associated with chronic disease.^27^

In terms of variation across index diagnoses, the out-of-pocket costs for ST elevation myocardial infarction are, on average, slightly higher than non-ST elevation myocardial infarction and unstable angina. This is related to differences in the complexity of treatments for these events and is reflected in the higher risk of catastrophic health expenditure in participants with ST elevation myocardial infarction relative to those with non-ST elevation myocardial infarction or unstable angina.

Out-of-pocket costs were lower in those with health insurance. Notably, in China, India, Thailand and Viet Nam, participants with health insurance have significantly lower risks of financial catastrophe than those without insurance. In China, although health insurance was protective, more than 50% of insured participants nonetheless incurred catastrophic health expenditure. This is most likely due to a lower threshold for financial catastrophe resulting from a combination of gaps in insurance coverage, high co-payments and low incomes. Such findings are consistent with a previous study in China, in which 53% (1798/3384) of stroke survivors who reported catastrophic health expenditure had health insurance.^13^ Current initiatives to roll out social insurance programmes in China – particularly to rural populations through rural cooperative medical schemes – may go some way to addressing this problem.^28^ However, the high costs of increasingly prevalent disease events associated with chronic noncommunicable diseases will continue to present challenges to policy-makers over coming years.

Given substantial differences in the types of health-care systems involved in this study, health insurance has different roles across the various settings. In health-care systems such as those used in Malaysia, the Republic of Korea, Singapore and Thailand, where there is universal health coverage, health insurance is largely supplementary cover. This may explain why, in the Republic of Korea for instance, there were higher rates of catastrophic health expenditure in insured compared to uninsured participants. Here, participants who are insured may be opting for care through the private system, for reasons such as the avoidance of waiting lists for non-urgent procedures, choice of provider and superior hotel services. They may incur private insurance co-payments and treatment costs not covered under their policies. In contrast, uninsured participants may have been receiving free or heavily subsidized services through the public system.

Financial catastrophe appears to increase with age up to 75 years, whereupon participants experience a lower risk. The greater complexity of illness with increasing age may increase costs associated with an event. The fact that this association is reversed in participants from the highest age groups may be due to higher mortality and an emphasis on more conservative treatment and palliation in this group.

There are three main limitations of the study. First, being clinic-based, the study assessed only the costs incurred by those participants who were able to afford treatment. The study therefore lacks an account of this aspect of financial burden where individuals are excluded from treatment due to cost. Second, only 73% of the participants were able to complete income and cost data. In some countries, participants were recruited from a few centres only. This limitation mirrors well-recognized difficulties in eliciting economic data^29^ and limits the generalizability of the findings. Third, the relatively small numbers of participants from countries other than China and India limited the ability to conduct adjusted analyses for individual countries. The study should not be viewed as a series of individual country sub-studies but a multi-country analysis. The fact that there were smaller numbers of participants enrolled in smaller countries does not detract from the generalizability of the findings since financial catastrophe – a standardized outcome indicator – was used.

Ensuring that treatment for acute coronary syndromes is provided and financed affordably in future years will be a major challenge. In particular, this will entail the funding of financial protection programmes that adequately offset the high costs of conditions such as acute coronary syndromes in low-resource settings. While these findings relate to the acute phase of hospitalization, the need to provide affordable, long-term treatment and rehabilitation presents further significant challenges.
